# Silk Fibroin/Collagen/Chitosan Scaffolds Cross-Linked by a Glyoxal Solution as Biomaterials toward Bone Tissue Regeneration

**DOI:** 10.3390/ma13153433

**Published:** 2020-08-04

**Authors:** Sylwia Grabska-Zielińska, Alina Sionkowska, Catarina C. Coelho, Fernando J. Monteiro

**Affiliations:** 1 Department of Physical Chemistry and Physicochemistry of Polymers, Faculty of Chemistry, Nicolaus Copernicus University in Toruń, 87-100 Toruń, Poland; 2 Department of Chemistry of Biomaterials and Cosmetics, Faculty of Chemistry, Nicolaus Copernicus University in Toruń, 87-100 Toruń, Poland; as@chem.umk.pl; 3 i3S—Instituto de Investigação e Inovação em Saúde, Universidade do Porto, 4200-135 Porto, Portugal; catarina.coelho@i3s.up.pt (C.C.C.); fjmont@i3s.up.pt (F.J.M.); 4 INEB—Instituto de Engenharia Biomédica, Universidade do Porto, 4200-180 Porto, Portugal; 5 FEUP—Faculdade de Engenharia, Universidade do Porto, 4200-465 Porto, Portugal; 6 FLUIDINOVA, S.A., 4470-605 Moreira da Maia, Portugal

**Keywords:** silk fibroin, collagen, chitosan, MG-63, three-component scaffolds, glyoxal solution

## Abstract

In this study, three-dimensional materials based on blends of silk fibroin (SF), collagen (Coll), and chitosan (CTS) cross-linked by glyoxal solution were prepared and the properties of the new materials were studied. The structure of the composites and the interactions between scaffold components were studied using FTIR spectroscopy. The microstructure was observed using a scanning electron microscope. The following properties of the materials were measured: density and porosity, moisture content, and swelling degree. Mechanical properties of the 3D materials under compression were studied. Additionally, the metabolic activity of MG-63 osteoblast-like cells on materials was examined. It was found that the materials were characterized by a high swelling degree (up to 3000% after 1 h of immersion) and good porosity (in the range of 80–90%), which can be suitable for tissue engineering applications. None of the materials showed cytotoxicity toward MG-63 cells.

## 1. Introduction

There is an increasing need for new biopolymeric materials for cell-based transplantation, gene therapy, and tissue engineering [1]. Materials based on polymers are used to obtain implants for filling small bone defects or cartilage tissue cavities [1,2]. Three-dimensional scaffolds supporting the regeneration of damaged tissue should perform the following functions: mechanical support for cells and proteins, merging the cells in the tissue, formation and movement of cells, and having an influence on the development and differentiation of cells. Three-dimensional materials should be characterized by a combination of mechanical, biological, and chemical properties [3,4,5]. One such feature is the porosity, whereby cells can penetrate deep into the material. This leads to cell proliferation and the reconstruction of tissues [6]. Increasingly, biopolymers are used for this type of biomaterial because they are biocompatible. Natural polymer-based biomaterials can be biodegradable. The implant slowly degrades while reconstituting the tissue. A number of protein polymers (collagen, gelatin, silk fibroin) and polysaccharides (hyaluronic acid, chitosan, sodium alginate, chondroitin sulphate, carrageenan) are used in biomaterials [7,8,9,10,11]. The large molecular compounds, derived from natural sources, are characterized by properties desired in tissue engineering: biocompatibility, biodegradability, and a lack of immune responses after introduction into the human body [12,13]. Currently, implants based on polymers are most commonly manufactured with chitosan and collagen [14,15,16]. They were studied in terms of the physico-chemical properties [17,18,19,20,21,22,23] and biological properties [24,25]. However, materials containing collagen exhibit poor stability in wet conditions and poor mechanical properties [26,27]. Therefore, it is necessary to search for new materials that can potentially be used in tissue engineering. For the production of biomaterials with better properties, materials based on mixtures of two or more biopolymers should be used [20,21,22]. Significantly better mechanical properties have been demonstrated for silk fibroin [28]. Silk fibroin is a protein composed of raw silk, where it performs structural functions [1].

Two-component mixtures based on natural polymers have been tested by our group [29,30,31,32,33,34,35,36,37,38,39,40]. Collagen/chitosan mixtures were used to create films and scaffolds [29,30,31,32]. Materials were characterized by porosity and good mechanical properties. Silk fibroin was mixed with collagen in ratios of 50/50, 25/75, and 75/25 to prepare biopolymer sponges [33,34,35,36]. Furthermore, silk fibroin and chitosan were previously studied by our group [37,38,39,40]. Nevertheless, we decided to further improve the properties of the previously obtained materials. We resolved to create ternary mixtures based on the three previously mentioned polymers (silk fibroin, collagen, and chitosan). Three types of blend mixtures were prepared: collagen (Coll)/chitosan (CTS), silk fibroin (SF)/Coll, and SF/CTS. To each of the mixtures in a 50/50 ratio, the third component was added in the amounts of 10, 20, and 30%.

In addition, these kinds of mixtures can be subjected to a cross-linking process to improve some parameters, such as the regularity of pores and stability in a wet environment. After the cross-linking process, biomaterials should not be affected by changes in biodegradability and biocompatibility. This type of modification is expected to improve the mechanical properties of materials, stability in wet conditions, and degradation resistance [23].

Glyoxal is a very simple organic compound with two aldehyde groups. It is the smallest dialdehyde. Glyoxal is a really common cross-linking agent of polysaccharides [41,42,43,44] and proteins [45,46]. Their reactions with glyoxal are very well documented. In the case of polysaccharides, regarding the reaction with chitosan, glyoxal has cross-linking ability via acetal formation between the aldehyde groups of glyoxal and the hydroxyl groups of the glucosamine units of chitosan, or through Schiff’s base formation between the free amino groups of chitosan and the aldehyde groups of glyoxal [47,48]. In the case of proteins, glyoxal is a reactive oxoaldehyde [45]. It has been reported to interact with several proteins, e.g., bovine serum albumin [49], α-crystallin [50], myoglobin [51,52], α-synuclein [53], and hemoglobin [54,55,56]. Glyoxal is capable of modifying lysine and arginine residues of proteins to form several AGE (advanced glycation end product) adducts, such as carboxymethylarginine, carboxymethyllysine, hydroimidazolones, and dihydroxyimidazolidines [45]. In the silk fibroin chain, several reactive amino acids are present. It allows for chemical modification of these amino acids via chemical reactions. Such chemical modification can be an attractive strategy for tailoring the protein to the desired bio-medical application [45]. As far as collagen is concerned, glyoxal is a known apoptosis-inducing agent that is involved in the formation of AGEs [46].

In this study, silk-fibroin-, collagen-, and chitosan-based scaffolds cross-linked by glyoxal were prepared and the properties of the scaffolds were examined. It is worth mentioning that 3D scaffolds based on blends of silk fibroin, collagen, and chitosan without any cross-linking can also be obtained, but the properties of such scaffolds are very far from the requirements for practical applications as biomaterials. As it may be seen in this work, the glyoxal solution was used to modify each of the biopolymer blends prepared in this study. The aim of this work was to observe the structure, porosity, density, swelling ratio, moisture content, mechanical properties, and cytotoxicity of glyoxal-solution-cross-linked materials based on ternary biopolymer blends. Glyoxal solution should be responsible for improving some physico-chemical properties of the material: swelling ability, density, porosity, and mechanical properties [44]. To our best knowledge, the use of glyoxal solution to modify a three-component scaffold is a novelty in biopolymer materials treatments. There are no earlier reports in the scientific literature about three-component mixtures cross-linked by this agent. There are a lot of reports about cross-linking two- and three-component mixtures by other agents: EDC/NHS (N-(3-dimethylaminopropyl)-N’-ethylcarbodiimide hydrochloride/N-hydroxysuccinimide) [57,58,59], dialdehyde starch [59,60,61], genipin [62,63], tannic acid [64,65], or dialdehyde chitosan [66,67]. It should be emphasized that glyoxal solution has some advantages, such as lower cost and easy transfer and application, as well as good adhesiveness [68].

## 2. Materials and Methods

The first biopolymer, chitosan (CTS) was supplied by Sigma-Aldrich (Poznań, Poland). The viscosity average molecular weight of chitosan was 0.59 × 10^6^ g/mol and the deacetylation degree (DD, %) was 78%. The second and third biopolymers were silk fibroin and collagen, respectively. They were obtained in our laboratory. Collagen was obtained from young rat tail tendons [69]. The tendons were excised and washed once in distilled water. After that, they were dissolved in 0.1 M acetic acid for three days at 4 °C. The solution was centrifuged (10 min at 10,000 rpm) to get rid of the undissolved parts. After centrifugation, the solution was frozen (−18 °C) and lyophilized (−55 °C, 5 Pa for 48 h, ALPHA 1–2 LD plus, CHRIST, Osterode am Harz, Germany). Silk fibroin was prepared from *Bombyx mori* cocoons (Jedwab Polski Sp. z o.o., Milanówek, Poland) according to a procedure described in the literature [60]. The cocoons were boiled in an aqueous solution of 0.5% Na_2_CO_3_ for 1 h, twice. Then, the cocoons were boiled in a 5% alkaline soap solution for 30 min. To extract the sericin proteins, the cocoons were boiled in distilled water for 20 min. It was repeated three times. Subsequently, the prepared silk fibroin was dried in room conditions (temperature and humidity). Then, the silk fibroin was dissolved in 9.3 M lithium bromide. The dissolution process was carried out at 80 °C for 4 h. Silk fibroin was prepared as a 5% concentrated solution. Then, the solution was filtered. Chitosan and collagen were prepared as a 1% solution in 0.1 M acetic acid.

Three types of mixtures were prepared (Table 1). The first type was chitosan and collagen (50/50 weight ratio) mixtures with a 10, 20, and 30% silk fibroin addition. The second type was silk fibroin and collagen (50/50 weight ratio) mixtures with a 10, 20, and 30% chitosan addition, and the third type was 50/50 weight ratio silk fibroin/collagen mixtures with a 10, 20, and 30% chitosan addition. The biopolymers were mixed together with the use of a magnetic stirrer for 3 h. The mixtures were cross-linked by a glyoxal solution. The cross-linking agent was used as a 5% addition relative to the weight of the polymers in the mixture [70,71]. The three-component mixtures were mixed with the cross-linking agent with the use of a magnetic stirrer for 1 h. After mixing, the mixtures were dialyzed against distilled water for 3 days to aqueous solutions. Then, the mixtures were poured into 24-well polystyrene culture plates, frozen (−80 °C, 24 h), and lyophilized (−55 °C, 5 Pa, 48 h, ALPHA 1–2 LDplus, CHRIST, Osterode am Harz, Germany).

### 2.1. Fourier-Transform Infrared Spectroscopy (FTIR)

The changes in the chemical structure of the mixtures were evaluated via FTIR spectroscopy using a Nicolet iS10 spectrophotometer equipped with an ATR device with a diamond crystal (Thermo Fisher Scientific, Waltham, MA, USA). The 64 scans were collected with a resolution of 4 cm^−1^ in the range of 400–4000 cm^−1^.

### 2.2. Density and Porosity

Density and porosity were measured using the liquid displacement method with isopropanol as the liquid. Isopropanol was used because it did not dissolve the samples [60,72]. Samples of each type were measured in triplicate. A porous scaffold with a known weight (*W*) was immersed for 3 min in a cylinder with a known volume of isopropanol (*V*_1_). The volume of isopropanol with the scaffold was measured (*V*_2_) and the volume of liquid after the removal of sample (*V*_3_) was measured [60]. The density of the scaffold (*d*) was calculated using Equation (1), and the porosity (*ε*) was calculated using Equation (2):

(1) $$d=\frac{W}{V_{2}-V_{3}}\;\left(\mathrm{in}\;\frac{\mathrm{mg}}{{\mathrm{cm}}^{3}}\right)\;$$

(2) $$\varepsilon =\frac{V_{1}-V_{3}}{V_{2}-V_{3}}\times 100\%$$

V_1_—initial volume of isopropanol (cm^3^),
*V*_2_—total volume of isopropanol and isopropanol impregnated sample (cm^3^),
*V*_3_—isopropanol volume after scaffold removal (cm^3^),
*W*—sample weight (mg).

### 2.3. Moisture Content and Swelling Behavior

The scaffold moisture content was measured by drying samples in an oven (DZ-2BC Vacuum Oven, ChemLand, Stargard, Poland) at 105 °C until they reached a constant weight. The weighing results were expressed as grams of water in 100 g of dry sample weight [60]. Samples of each type were measured in triplicate.

The swelling ratio was evaluated by immersing the composites’ fragments in 15 mL of PBS (phosphate-buffered saline) solution, pH = 7.4 at 37 °C (incubator, Sanyo, Japan). After 1 h, 2 h, 4 h, 1 day, 3 days, and 7 days of immersion, the samples were gently dried by putting them between two sheets of paper and then weighted (analytical balance, OHAUS PA114CM/1, Europe GmbH, Greifensee, Switzerland) [60,73]. The swelling ratios were found using Equation (3):

(3) $$\mathrm{swelling}=\frac{m_{t}-m_{0}}{m_{0}}\times 100\%\;$$

*m_t_*—weight of the sample after immersion in PBS (mg),
*m*_0_—weight of the sample before immersion (mg).
Samples of each type were measured in triplicate.

### 2.4. Microstructure of the Scaffolds

The microstructure of the materials was observed using a scanning electron microscope (SEM) (LEO Electron Microscopy Ltd., Cambridge, England, UK). The samples were frozen in liquid nitrogen (3 min). This was done because freezing a scaffold allows for its gentle cutting with a razor scalpel to observe its interior microstructure [60,74]. The samples were covered with gold and scanning electron microscope images were made with 200 and 500 μm resolutions. The samples’ microstructure before and after immersion in PBS was compared.

### 2.5. Mechanical Properties

Mechanical properties of the materials were studied using a Zwick&Roell 0.5 testing machine (Zwick&Roell Group, Ulm, Germany) with the crosshead speed set at 0.5 mm/min in accordance with the Polish Norm PN-81/C-89034 (ISO 527-1 i 527-2) standard procedure [60]. The diameter of the samples was 14.44 mm, with the thickness in the range of 13.0–14.9 mm.

Mechanical properties of all the scaffolds were studied in room conditions (temperature and humidity) and in wet conditions (water-PBS). Five samples of each type were studied. The scaffold was placed between two discs and compressed [60]. The compressive strength, Young’s modulus, and the strength of the 0.2% plastic deformation were then evaluated.

### 2.6. In Vitro Cytotoxicity Assay—Metabolic Activity

The cytotoxicity of the scaffolds was tested using MG-63 osteoblast-like cells (MG63, ATCC, Manassas, VA, USA). With that purpose in mind, an Alamar Blue^®^ (resazurin) assay was used to assess the metabolic activity. Resazurin is a blue dye that is reduced in mitochondria to the pink-colored and highly red fluorescent resorufin.

A human bone osteosarcoma cell line (MG63, ATCC) was cultured in α-MEM (alpha modification of Eagle minimum essential medium, Sigma-Aldrich, St. Louis, MO, USA), supplemented with 10% fetal bovine serum (FBS, Gibco, Thermo Fisher Scientific, Waltham, MA, USA), 1% penicillin-streptomycin (3 × 10^−4^ mol/L and 5 × 10^−4^ mol/L, Gibco), and maintained at 37 °C and 5% carbon dioxide (CO_2_). Prior to the cell culture, the scaffolds (2–4 mm in height, 14.44 mm in diameter) were sterilized by soaking them in 70% ethanol aqueous solution. After that, they were washed five times with sterile PBS (pH = 7.4, Sigma-Aldrich, St. Louis, MO, USA). After the cell confluence, the cells were seeded via a drop method onto scaffolds. Cells were seeded on 24-well plates using a cell density of 1 × 10^4^ cells/mL per sample and allowed to adhere at 37 °C. The cells seeded directly on tissue culture polystyrene (TCPS, Thermo Fisher Scientific, Waltham, MA, USA) served as a control. The analysis was run in triplicate [60].

Resazurin with a 10% (*v*/*v*) concentration was added to the medium and incubated for 4 h at 37 °C and 5% CO_2_. Afterward, 100 µL of resazurin was transferred into a 96-well black plate. The fluorescence was measured at 530 nm excitation and 590 nm emission wavelength with a fluorescence reader (SynergyMix, BioTek, Winooski, VT, USA) using Gen5 1.09 Data Analysis Software (BioTek, Winooski, VT, USA). The results were expressed as relative fluorescent units (RFU) for each time point. These measurements were made at 1, 3, and 7 days of culture [60]. Samples of each type were measured in triplicate.

### 2.7. Statistical Analysis

Statistical analysis was completed using commercial software (SigmaPlot 14.0, Systat Software, San Jose, CA, USA). To assess the normal distribution of the data, the Shapiro–Wilk test was used. Results were presented as a mean ± standard deviation (SD) and were statistically analyzed using one-way analysis of variance (one-way ANOVA). Multiple comparisons between means were performed using the Bonferroni t-test with the statistical significance set at *p* < 0.05.

## 3. Results and Discussion

### 3.1. Fourier-Transform Infrared Spectroscopy Spectroscopy (FTIR)

**Figure 1:**
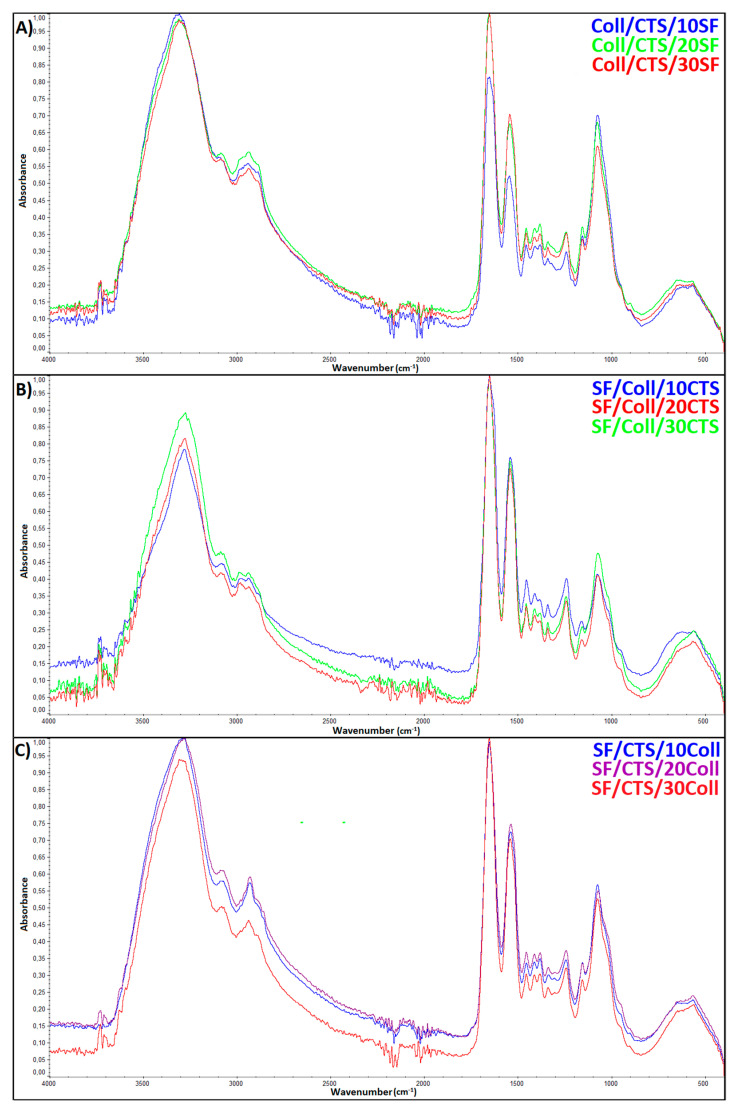
The ATR-FTIR spectra of the three-component scaffolds cross-linked by glyoxal solutions: (**A**) Coll/CTS mixture with 10, 20, and 30% SF; (**B**) SF/Coll mixture with 10, 20, and 30% CTS; and (**C**) SF/CTS with 10, 20 and 30% Coll.

The structure of the three-component scaffolds based on silk fibroin, collagen, and chitosan cross-linked by a glyoxal solution were characterized using attenuated total reflection infrared spectroscopy (ATR-FTIR). The ATR-FTIR spectra are shown in Figure 1. From the FTIR spectra, the characteristic bands of the scaffolds cross-linked by a glyoxal solution may be seen. The wide band at ≈3400 cm^−1^ was due to O-H and N-H stretching vibrations and the band at ≈2800 cm^−1^ was due to C-H stretching vibrations in -CH and -CH_2_ groups [44,75]. A characteristic band of the amide group amide I was observed at ≈1650 cm^−1^. A band at ≈1600 cm^−1^ was responsible for the -NH_2_ bending vibrations. Other bands could also be observed in the spectra: at ≈1420 cm^−1^, -NH_2_ bending vibration of the primary amino group; at ≈1380 cm^−1^, -CH_3_ symmetric deformation; at ≈1320 cm^−1^, C-N stretching vibrations; and at ≈1260 cm^−1^, characteristic amide III stretching vibrations [44]. For the chitosan-based scaffold after the crosslinking reaction, a new peak appeared at ≈1110 cm^−1^. This can indicate that glyoxals have reacted with the hydroxyls of the glucosamine rings due to acetalization [48]. At ≈1050 cm^−1^, C-O-C stretching vibrations peak due to the pyranose ring was observed [44]. There were no significant band position changes between the three types of mixtures (relative to the Coll/CTS, SF/Coll, and SF/CTS based materials) to be observed (Figure 1). However, changes in the intensity of the bands could be noticed.

### 3.2. Density and Porosity

**Figure 2:**
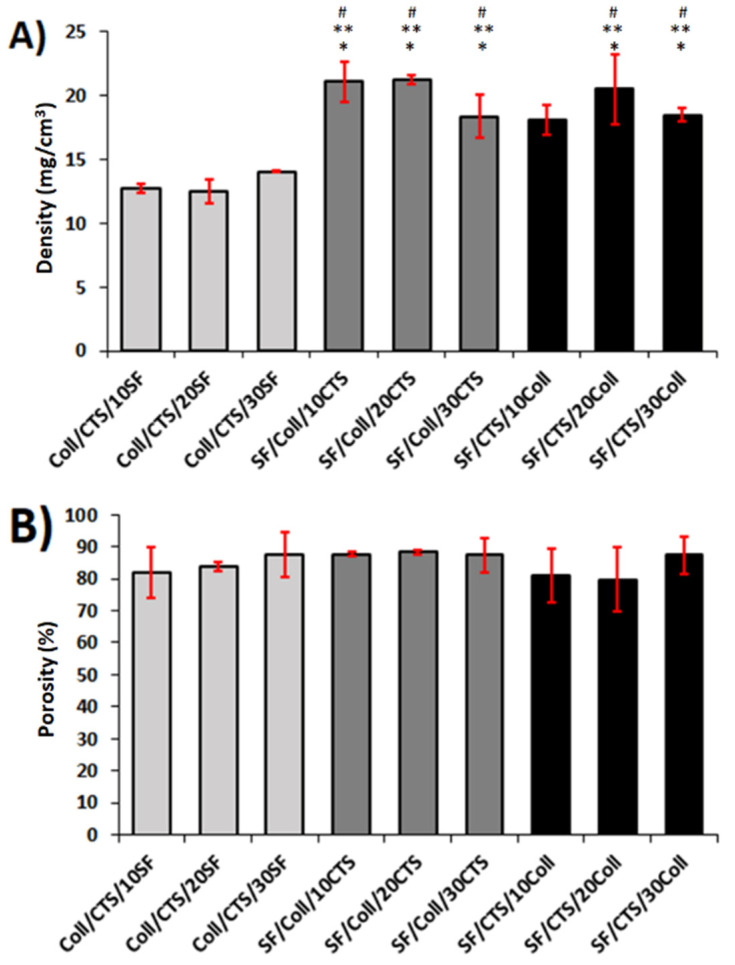
The density (**A**) and porosity (**B**) of 3D scaffolds (*n* = 3, mean ± SD, * significantly different vs. Coll/CTS/10SF, ** significantly different vs. Coll/CTS/20SF, # significantly different vs. Coll/CTS/30SF, *p* < 0.05).

Density and porosity are very important parameters from the point of view of biomedical applications of materials [76]. These parameters have to be defined for porous materials intended for tissue engineering applications [60]. The porosity and density were studied for the three-component materials with a cross-linking agent (GS) and their results are presented in Figure 2. The highest densities of the materials were observed for SF/Coll/10CTS (21.11 ± 1.56 mg/cm^3^) and SF/Coll/20CTS (21.25 ± 0.32 mg/cm^3^). The group of materials based on the 50/50 SF/CTS mixture with the addition of collagen was characterized by the highest density (18.38–21.25 mg/cm^3^). A slightly lower density was observed for materials based on the 50/50 SF/Coll mixture with the addition of chitosan (18.13–20.53 mg/cm^3^). The group of materials based on the 50/50 SF/CTS mixture with the addition of collagen presented the lowest density (12.56–14.05 mg/cm^3^). An increased matrix density enhances cell proliferation due to an increase in matrix stiffness. Increasing the density of the scaffold would favor proliferation and cell growth, as more scaffolds can be accessed by the cells [77,78].

It is commonly known that the appropriate porosity of the scaffold for bone tissue regeneration should be about 80–90% [77,78]. This porosity provides enough space for cell attachment and proliferation and allows for sufficient nutrient and gas exchange. A similar relationship was observed for both porosity and density: materials based on the 50/50 SF/CTS mixture with the addition of collagen were characterized by the highest porosity (87–90%). However, it should be mentioned that there were no significant differences between the several studied materials. The porosity for all the cross-linked scaffolds was in the range of 80–90%.

### 3.3. Moisture Content and Swelling Behaviour

**Figure 3:**
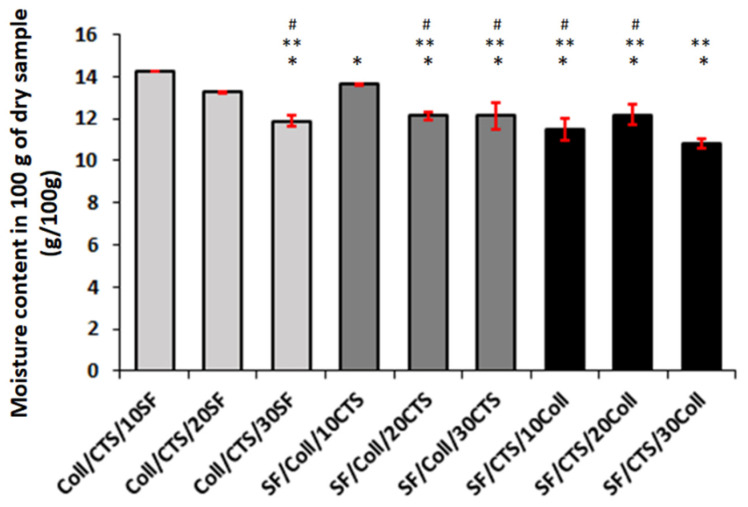
Results for the moisture content measurements of three component materials cross-linked by glyoxal solution (*n* = 3, mean ± SD, * significantly different vs. Coll/CTS/10SF, ** significantly different vs. Coll/CTS/20SF, # significantly different vs. Coll/CTS/30SF, *p* < 0.05).

The moisture content of scaffolds is shown in Figure 3. The results were expressed as grams of water in 100 g of dry sample weight. The highest moisture content was observed for Coll/CTS/10SF scaffolds (14.29 g/100 g), while the lowest was for the SF/CTS/30Coll sample (only 10.84 ± 0.22 g/100 g). These are similar results to those in Coll (14.60 g/100 g) [59] and slightly lower results in our previously published report for Coll (13.30 ± 0.79 g/100 g), SF/Coll (17.33 ± 0.30 g/100 g), and SF/Coll/CTS (19.60 ± 1.35 g/100 g) cross-linked by dialdehyde starch [60]. However, the moisture content for all the samples was on a similar level (in the range of 10.84 to 14.29 g/100 g).

**Figure 4:**
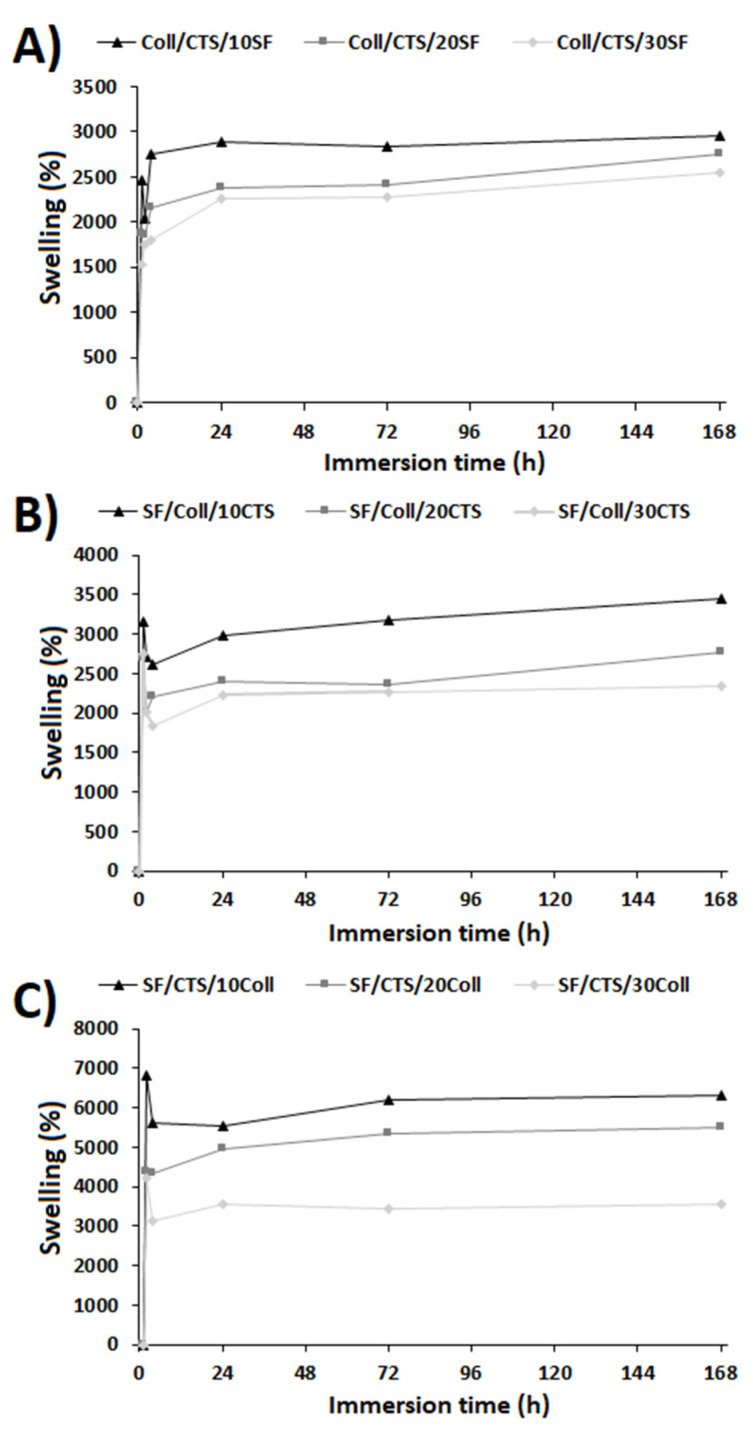
Swelling degree of the three-component materials cross-linked by glyoxal solution: (**A**) Coll/CTS mixture with 10, 20, and 30% SF; (**B**) SF/Coll mixture with 10, 20, and 30% CTS; and (**C**) SF/CTS with 10, 20, and 30% Coll (*n* = 3).

The swelling behavior for the studied materials is shown in Figure 4. The measurements were carried out for 7 days at 5 time points: 1 h, 2 h, 24 h, 72 h, 168 h. The high swelling degree is the characteristic property of hydrophilic and porous materials [60]. The behavior of the material after its application inside the body can be studied by immersing the materials in PBS solution (phosphate-buffer saline) at pH = 7.4 (relevant to the pH of blood) [60,72,73]. Thanks to the presence of a large number of functional groups capable of binding water, the materials containing silk fibroin, collagen, and chitosan are easily wettable by polar solvents and exhibit a very high swelling ability [72,73]. Swelling behavior is dependent on the material’s composition. With the increase of the third component addition to binary scaffolds, the swelling degree decreased. The lowest swelling degree, after each timepoint measurement, could be seen for the SF/Coll/30CTS sample. SF/CTS/10Coll material after 1 h of immersion was characterized by the highest swelling degree (6801 ± 94%). After 2 h of immersion, the swelling degree was slightly lower and stable. The swelling ability of each kind of sample began to stabilize after the second (2 h) or third (24 h) timepoint. The results of the analysis indicated that the three-component samples were characterized by a very high water absorption capacity, higher than materials made of one or two components. This observation is in good agreement with other research on binary and ternary blends [60,64,70,71].

### 3.4. Microstructure of the Scaffolds

**Figure 5:**
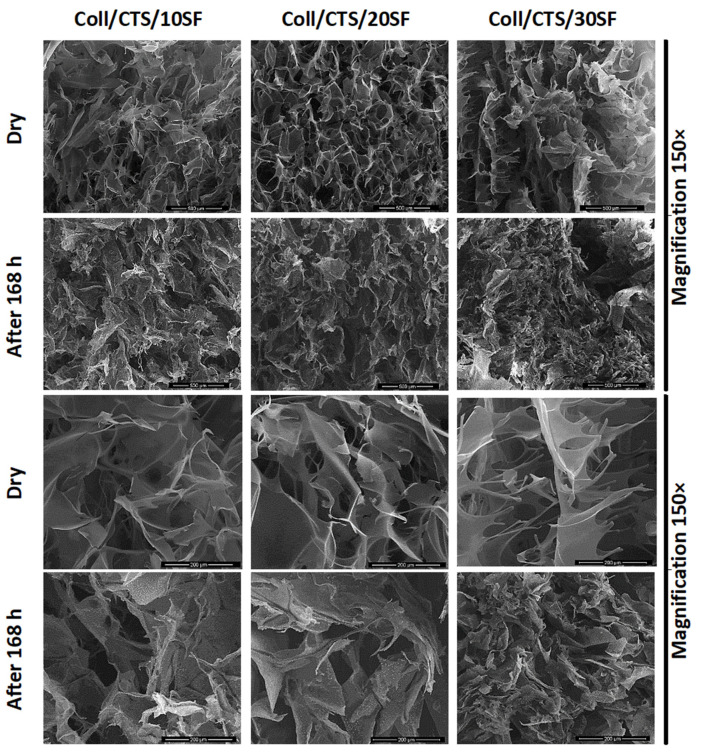
SEM images (magnification 500× and 150×) of Coll/CTS mixtures with 10, 20, and 30% SF (left to right columns) for dry scaffolds and after 168 h of immersion in PBS.

**Figure 6:**
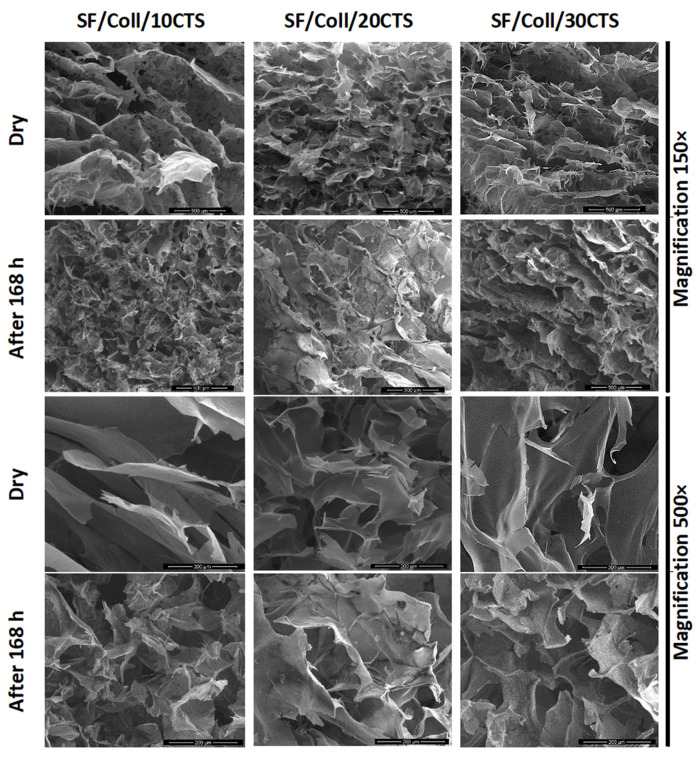
SEM images (magnification 500× and 150×) of SF/Coll mixtures with 10, 20, and 30% CTS (left to right columns) for dry scaffolds and after 168 h of immersion in PBS.

**Figure 7:**
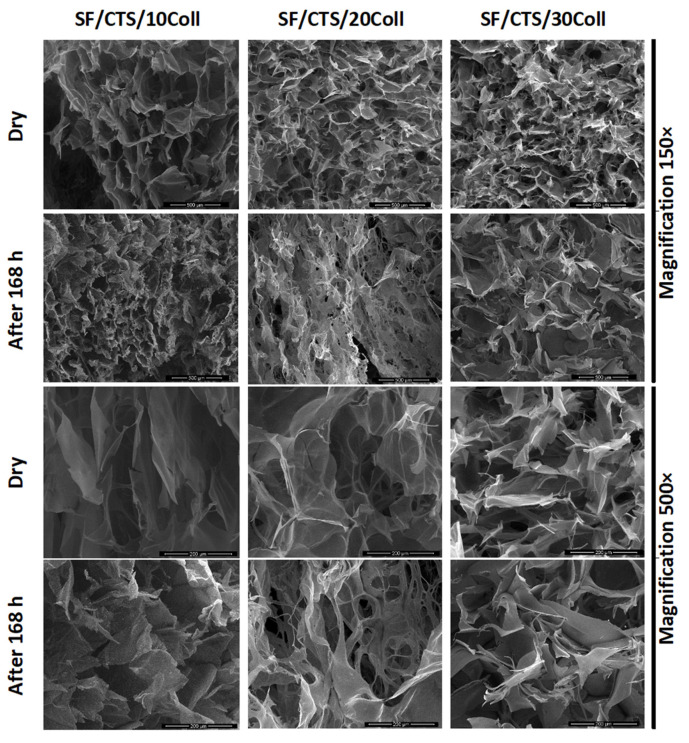
SEM images (magnification 500× and 150×) of SF/CTS mixtures with 10, 20 and 30% Coll (left to right columns) for dry scaffolds and after 168 h of immersion in PBS.

The microstructure of the scaffolds before immersion in PBS and after 168 h of immersion was studied using scanning electron microscopy (Figure 5, Figure 6 and Figure 7). The resolutions used in this study were 200 µm and 500 µm. After immersion in PBS, the scaffolds were dried for 7 days at room temperature and humidity. Each scaffold obtained in our study had a porous structure with interconnected pores. Such a material microstructure is required for tissue engineering applications [72,79]. Based on the SEM images, the size of the pores was determined [80,81]. It can be seen that the pores were smaller than 200 µm. In agreement with Hulbert et al., the minimum pore size required for bone tissue regeneration is generally considered to be ≈100 μm [82]. Nevertheless, larger pores (100–200 μm) showed substantial bone ingrowth as well. The larger pore size allows a greater number of blood vessels to grow [80]. The pores of the studied materials had similar sizes to the pores in scaffolds made of collagen, hyaluronic acid, and chitosan [83], and to materials made of silk fibroin/collagen with a 25% addition of chitosan cross-linked by dialdehyde starch, according to our previous report [60]. However, no significant changes were observed for different composition scaffolds based on ternary mixtures, as in our earlier research, when collagen and chitosan were mixed with sodium alginate [32] and when chitosan and collagen were mixed with hyaluronic acid [79,84]. The degradation of different sample types was observed using SEM. The scaffold pores’ diameter was smaller when compared to that of the scaffolds with a dry surface. The pores were squeezed. The images show that the erosion process resulting from the PBS salt presence was not initiated during the immersion of all the scaffold types. The structural differences resulted from swelling and consecutive drying.

### 3.5. Mechanical Properties

**Figure 8:**
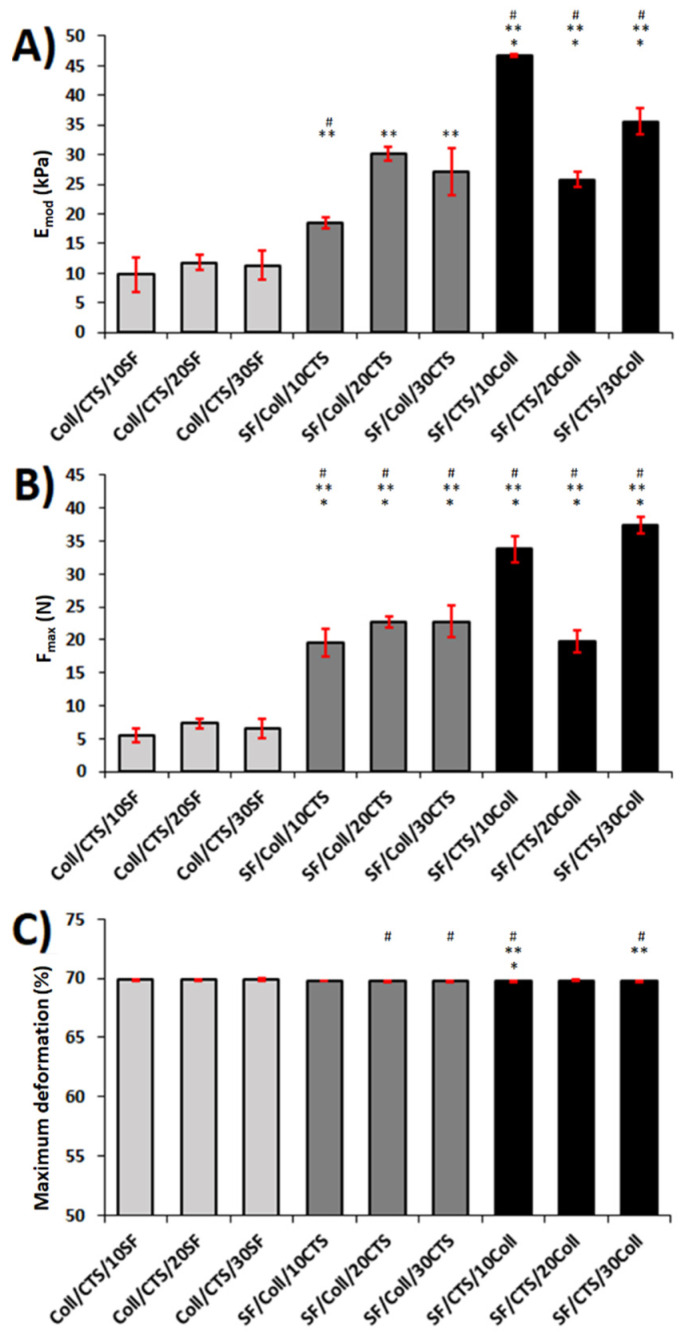
Results ((**A**) Young’s modulus, (**B**) maximum force, and (**C**) maximum deformation) of the mechanical testing in room conditions (temperature and humidity) for each kind of scaffold (*n* = 5, mean ± SD, significantly different vs. Coll/CTS/10SF, * significantly different vs. Coll/CTS/20SF, # significantly different vs. Coll/CTS/30SF, *p* < 0.05).

**Figure 9:**
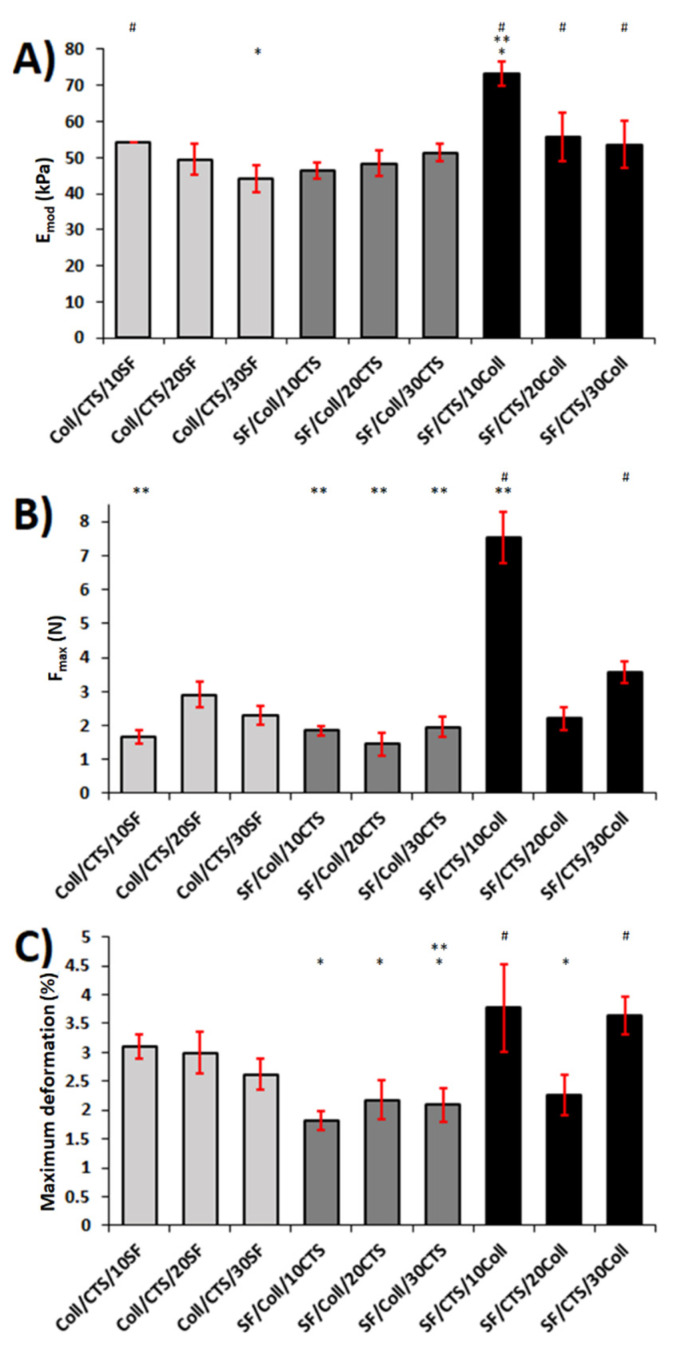
Results ((**A**) Young’s modulus, (**B**) maximum force and (**C**) maximum deformation) of mechanical testing of samples immersed in wet conditions (PBS solution) for each kind of scaffold (*n* = 5, mean ± SD, significantly different vs. Coll/CTS/10SF, * significantly different vs. Coll/CTS/20SF, # significantly different vs. Coll/CTS/30SF, *p* < 0.05).

Mechanical properties of the samples were tested in room conditions (temperature and humidity), as well as in wet conditions (water-PBS). Mechanical parameters of the scaffolds, namely, Young’s modulus (E_mod_), maximum force (F_max_), and maximum deformation, were determined and compared. The results of the mechanical testing in room conditions are shown in Figure 8. The results of the mechanical testing in wet conditions are shown in Figure 9. The value of Young’s modulus gives information about the elasticity and rigidity of the samples [69]. As the Young’s modulus increases, the material becomes more rigid. In room conditions, materials based on the SF/Coll and SF/CTS mixtures had higher Young’s moduli than the Coll/CTS based materials. The highest Young’s modulus among these scaffolds was observed for the SF/CTS/10Coll scaffold (46.7 ± 0.3 kPa). The lowest Young’s modulus was observed for the Coll/CTS/10SF material (9.7 ± 2.9 kPa). Materials based on Coll/CTS mixtures were characterized by the lowest rigidity. In wet conditions, the Young’s modulus was in the range of 44.1 ± 3.8 to 73.1 ± 3.4 kPa. The highest rigidity was observed for the SF/CTS/10Coll sample (73.1 ± 3.4 kPa). The materials based on the 50/50 SF/CTS blend with the addition of collagen were characterized by the best mechanical properties in room conditions, while the materials based on the 50/50 Coll/CTS blend with the addition of silk fibroin were characterized by the lowest mechanical properties. The SF/CTS-based materials were more rigid than the SF/Coll- and Coll/CTS-based materials. This means that a higher maximum strain had to be applied to compress the scaffold. The maximum deformation of the scaffold is a parameter that shows by what percentage the material was deformed when the maximum force was applied. There were no significant differences between each kind of scaffold in terms of the maximum deformation in the dry state. It was always above 70%. In wet conditions, the same behavior as in the dry state was observed. The best mechanical properties were observed for the 50/50 SF/CTS-based materials with the addition of collagen. Furthermore, in these conditions, the SF/CTS/10Coll scaffolds had the highest Young’s modulus (73.1 ± 3.4 kPa). The Coll/CTS- and SF/Coll-based materials were characterized by presenting a similar degree of compressive strength in the range of 44.1–54.0 kPa. The highest maximum force was observed for the SF/CTS/10Coll scaffold. In fact, this material was the most resistant to compression, both in dry and wet conditions. The maximum deformation values for materials dipped in water were not as stable as when in a dry state. The highest maximum deformation was observed for the SF/CTS/10Coll and SF/CTS/30Coll scaffolds, with 3.8 ± 0.3% and 3.6 ± 0.3%, respectively. Materials based on the 50/50 SF/Coll blend with a chitosan addition were characterized by the lowest maximum deformation, ranging between 1.8 ± 0.4% and 2.2 ± 0.5%.

### 3.6. In Vitro Cytotoxicity Assay—Metabolic Activity

**Figure 10:**
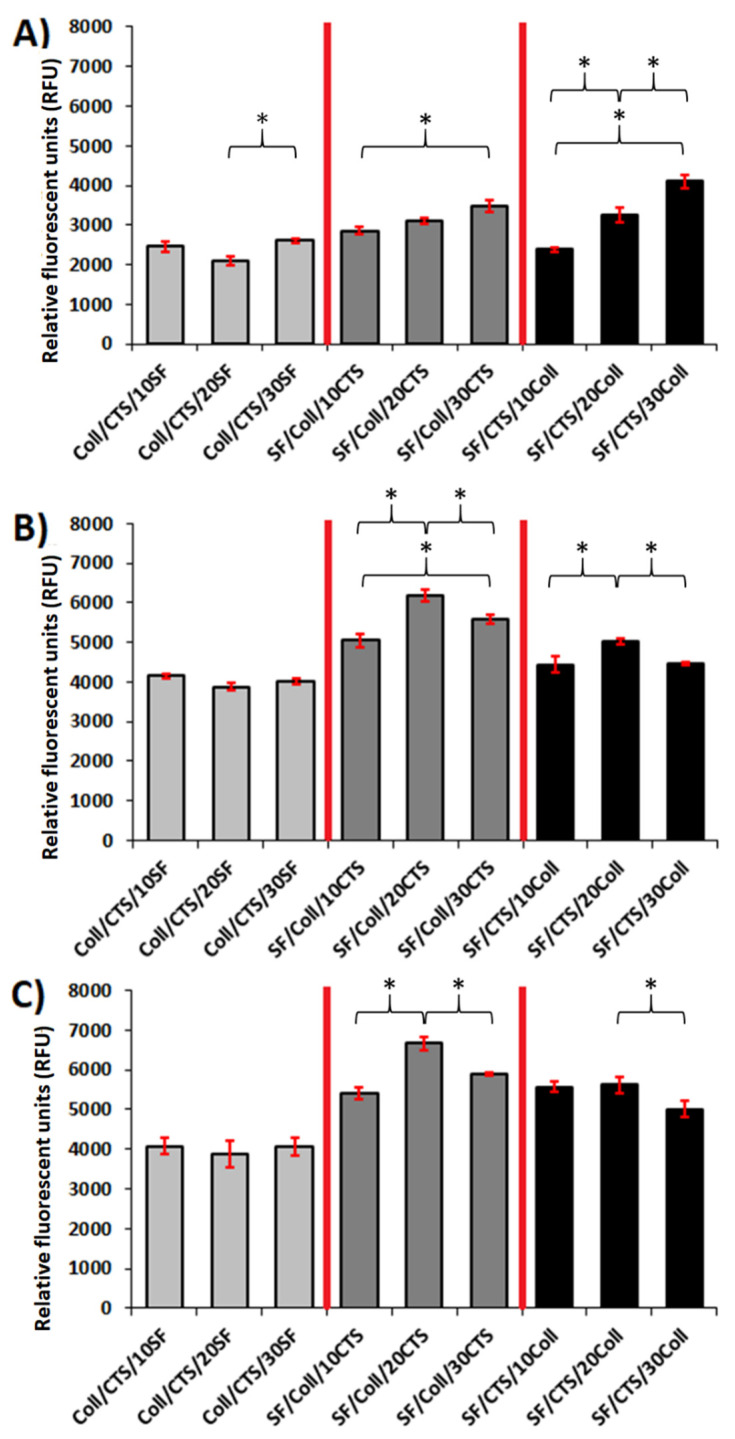
Metabolic activity after (**A**) 1 day, (**B**) 3 days, and (**C**) 7 days of MG-63 cells cultured on the studied scaffolds (*n* = 3, mean ± SD, * significantly different between the groups, *p* < 0.05).

The scaffolds developed in this work were designed to be implanted in small bone tissue defects. Scaffolds for tissue engineering have to be non-toxic [60,85]. To evaluate the cytotoxicity effect, a metabolic activity assay with MG-63 osteoblast-like cells was assessed [85]. The cell metabolic activity, evaluated using the resazurin assay and tested after 1, 3, and 7 days, is represented in Figure 10. This is a very useful test to assess mitochondrial metabolic activity, as the irreversible reaction of resazurin to resorufin is proportional to aerobic respiration [86]. Three types of samples were tested, namely, the 50/50 Coll/CTS, 50/50 SF/Coll, and 50/50 SF/CTS mixtures cross-linked by glyoxal solution, as the samples without cross-linking agent were very quickly dissolved. Therefore, the use of glyoxal as a crosslinker significantly increased the stability of the samples in wet conditions. For each type of sample, the metabolic activity of the cells increased as a function of the culture time. After the first day of cell culture, the metabolic activity of all the material types was at a similar level. The difference between the scaffold types was noticeable after the third day. SF/Coll-based materials proved to be a better construct for cells than the other two material types (Coll/CTS-based and SF/CTS-based materials). The higher metabolic activity was observed for scaffolds based on an SF/Coll mixture with a chitosan addition. It can be concluded that this type of material ensured a better environment for the interaction with cells among all the blends examined in this study.

To conclude, the analyzed samples were cytocompatible with MG-63 cells and it was observed that the metabolic activity increased over time. It can be assumed that cells cultured on these types of materials proliferated normally and colonized all the available surface of the material.

There was no toxic effect of the cross-linking agent observed in this study. Glyoxal solution can be a good alternative to other common cross-linkers, such as glutaraldehyde or formaldehyde, which may be harmful due to their possible toxicity [87]. There is also the possibility to use other cross-linking agents that give a similar effect to that found in this study, e.g., starch dialdehyde [59] or genipin [62]. However, glyoxal is a much more affordable cross-linker than the others, which makes it an appealing solution for the production of scaffolds with a lower cost and without compromising their properties.

## 4. Conclusions

Silk fibroin, collagen, and chitosan can be mixed and lyophilized to obtain materials in a 3D scaffold form. Glyoxal solution was a good cross-linking agent for three-dimensional materials based on the blends of silk fibroin, collagen, and chitosan. Cross-linking with glyoxal solution improved the materials’ properties at a low cost. It was found that cross-linked materials were characterized by a high swelling rate (up to 3000% after 1 h of immersion) and adequate porosity (in the range of 80 to 90%), which can be suitable for tissue engineering purposes. Mechanical parameters vary depending on the blend’s composition. The highest Young’s modulus among the studied scaffolds was observed for the SF/CTS/10Coll scaffold. None of the studied materials was cytotoxic to MG-63 cells. The most adequate scaffold for cell cultures was the one based on the two-component SF/Coll 50/50 mixture with a 20% chitosan addition. The cross-linking of ternary biopolymer blends with glyoxal may be a new way of modifying the materials, which offers a cheaper alternative to the existing methods of chemical cross-linking. It can be assumed that the obtained scaffolds can find potential biomedical applications in bone tissue regeneration.

## Figures and Tables

**Table 1:** The materials’ abbreviations and their explanations.

Abbreviation	Explanation
Coll/CTS	Mixture of collagen and chitosan in a 50/50 ratio
Coll/CTS/10SF	Mixture of collagen and chitosan in a 50/50 ratio, with a 10% addition of silk fibroin
Coll/CTS/20SF	Mixture of collagen and chitosan in a 50/50 ratio, with a 20% addition of silk fibroin
Coll/CTS/30SF	Mixture of collagen and chitosan in a 50/50 ratio, with a 30% addition of silk fibroin
SF/Coll	Mixture of silk fibroin and collagen in a 50/50 ratio
SF/Coll/10CTS	Mixture of silk fibroin and collagen in a 50/50 ratio, with a 10% addition of chitosan
SF/Coll/20CTS	Mixture of silk fibroin and collagen in a 50/50 ratio, with a 10% addition of chitosan
SF/Coll/20CTS	Mixture of silk fibroin and collagen in a 50/50 ratio, with a 10% addition of chitosan
SF/CTS	Mixture of silk fibroin and chitosan in a 50/50 ratio
SF/CTS/10Coll	Mixture of silk fibroin and chitosan in a 50/50 ratio, with a 10% addition of collagen
SF/CTS/20Coll	Mixture of silk fibroin and chitosan in a 50/50 ratio, with a 20% addition of collagen
SF/CTS/30Coll	Mixture of silk fibroin and chitosan in a 50/50 ratio, with a 30% addition of collagen
